# Echium oil is not protective against weight loss in head and neck cancer patients undergoing curative radio(chemo)therapy: a randomised-controlled trial

**DOI:** 10.1186/1472-6882-14-382

**Published:** 2014-10-07

**Authors:** Lies Pottel, Michelle Lycke, Tom Boterberg, Hans Pottel, Laurence Goethals, Fréderic Duprez, Alex Maes, Stefan Goemaere, Sylvie Rottey, Imogen Foubert, Philip R Debruyne

**Affiliations:** Cancer Centre, General Hospital Groeninge, Loofstraat 43, B-8500 Kortrijk, Belgium; Department of Radiation Oncology, Ghent University Hospital, Ghent, Belgium; Department of Public Health and Primary Care, Catholic University Leuven Kulak, Kortrijk, Belgium; Department of Nuclear Medicine, General Hospital Groeninge, Kortrijk, Belgium; Department of Osteoporosis and Metabolic Bone Diseases, Ghent University Hospital, Ghent, Belgium; Department of Medical Oncology, Ghent University Hospital, Ghent, Belgium; Department of Microbial and Molecular Systems, Food Science and Nutrition Research Centre (LForCe), Catholic University Leuven Kulak, Kortrijk, Belgium; Ageing and Cancer Research Cluster, Centre for Positive Ageing, University of Greenwich, London, UK

**Keywords:** Head and neck cancer, Cachexia, Echium oil, Weight loss, Radio(chemo)therapy, Curative intent

## Abstract

**Background:**

Therapy-induced mucositis and dysphagia puts head and neck (H&N) cancer patients at increased risk for developing cachexia. Omega-3 fatty acids (*n*-3 FA) have been suggested to protect against cachexia. We aimed to examine if echium oil, a plant source of *n*-3 FA, could reduce weight loss in H&N cancer patients undergoing radio(chemo)therapy with curative intent.

**Methods:**

In a double-blind trial, patients were randomly assigned to echium oil (intervention (I) group; 7.5 ml *bis in die* (b.i.d.), 235 mg/ml α-linolenic acid (ALA) + 95 mg/ml stearidonic acid (SDA) + 79 mg/ml γ-linolenic acid (GLA)) or *n*-3 FA deficient sunflower oil high oleic (control (C) group; 7.5 ml b.i.d.) additional to standard nutritional support during treatment. Differences in percentage weight loss between both groups were analysed according to the intention-to-treat principle. Erythrocyte FA profile, body composition, nutritional status and quality of life were collected.

**Results:**

Ninety-one eligible patients were randomised, of whom 83 were evaluable. Dietary supplement adherence was comparable in both groups (median, I: 87%, C: 81%). At week 4, the I group showed significantly increased values of erythrocyte *n*-3 eicosapentanoic acid (EPA, 14% vs −5%) and *n*-6 GLA (42% vs −20%) compared to the C group, without a significant change in *n*-6 arachidonic acid (AA, 2% vs −1%). Intention-to-treat analysis could not reveal a significant reduction in weight loss related to echium oil consumption (median weight loss, I: 8.9%, C: 7.6%). Also, no significant improvement was observed in the other evaluated anthropometric parameters.

**Conclusions:**

Echium oil effectively increased erythrocyte EPA and GLA FAs in H&N cancer patients. It failed however to protect against weight loss, or improve nutritional parameters.

**Trial registration:**

ClinicalTrials.gov Identifier NCT01596933.

## Background

Head and neck (H&N) cancer patients are a nutritionally vulnerable group because the causative factors alcohol and nicotine abuse are closely associated with nutritional deficiencies. Moreover, tumour localization and the intensive treatment may induce additional problems (acute dysphagia, nausea, altered taste perception, etc.) resulting in reduced food intake and progressive weight loss [1, 2]. More than half of the patients are at risk of malnutrition at time of treatment presentation, and the majority continues to lose weight during radiation treatment, with a mean weight loss of approximately 10% of their body weight [3, 4]. Prolonged weight loss can, in severe cases, result into cancer cachexia, a multifactorial wasting syndrome characterized by anorexia, an ongoing loss of skeletal muscle (with or without loss of fat mass), neuroendocrine dysfunction and chronic inflammation [5]. The true incidence of cachexia in patients with H&N cancer is unknown, but it has been observed in 80% of patients suffering from advanced cancer, causing death in 20% to 40% of these patients [6]. Several attempts to improve nutritional status of cancer patients by conventional oral supplements or parenteral nutrition, were mostly unsuccessful [7, 8]. Recently, long-chain omega-3 polyunsaturated fatty acids (*n*-3 LCPUFA), especially eicosapentaenoic acid (EPA), have been suggested to exert anti-inflammatory properties, through which they could be effective in reversing cancer cachexia [9, 10]. Ryan and his team demonstrated a significantly attenuated stress response in the EPA group of surgical esophageal cancer patients, a population comparable to H&N cancer patients regarding the underlying mechanisms resulting into a cachectic state [11]. *N*-3 PUFA can only be ingested through the diet, and dietary uptake below recommended levels has been associated with several Western diseases, e.g. cardiovascular disease and several inflammatory diseases [12]. Preliminary data have suggested that nutritional intervention with fish oil supplementation, which is the main source of *n*-3 LCPUFA, in cancer patients may lead to weight gain, maintenance of lean body mass, improved performance status and increased appetite in ambulatory H&N cancer patients when administered peri- or postoperatively [13–15]. However, recent *in vitro* research suggested that a specific fatty acid component of fish oil might counteract the efficacy of certain chemotherapeutic agents [16]. Also, supplement tolerance is often moderate due to aversion for palatability and occurrence of gastro-intestinal problems. Moreover, the rapid decline of wild fish is forcing researchers to look for alternative sources of *n*-3 LCPUFA [17–19]. Echium oil is a plant-based alternative extracted from the seeds of *Echium plantagineum*, containing significant amounts of the *n*-3 PUFA α-linolenic acid (ALA) and stearidonic acid (SDA), as well as *n*-6 PUFA γ-linolenic acid (GLA) and has been suggested as a true alternative for vegetarians to benefit from the anti-inflammatory effects of LCPUFA [20]. SDA is the product of the rate-limiting Δ6-desaturase step and therefore more readily converted to EPA, than when consuming ALA (conversion rate 17-30% vs 0.2-8%) [21, 22]. Moreover, supplementation with SDA in combination with GLA has been suggested to work synergistically in increasing anti-inflammatory effects through increases in EPA and dihomo-GLA (DGLA) concentrations, and thereby preventing the accumulation of the pro-inflammatory arachidonic acid (AA) [23].

In this placebo-controlled, multicentre clinical trial, we primarily aimed to examine the effect of echium oil as an alternative source of *n*-3 PUFA, to reduce weight loss in H&N cancer patients undergoing curative radio(chemo)therapy. In addition, we studied the tolerance of echium oil, its effect on erythrocyte fatty acid profile, as well as changes in body composition, nutritional status, treatment-related toxicity and quality of life as secondary endpoints.

## Methods

### Patients

Patients, aged ≥ 18 years, with a histologically confirmed diagnosis of squamous cell carcinoma of the head and neck, eligible for curative primary or adjuvant radiotherapy, with or without systemic treatment were recruited. Tumours of the parotid gland, nasal cavity, paranasal sinuses and patients with cT1 cN0 cM0 glottic cancer were excluded. Other exclusion criteria were: distant metastases, another non-cured cancer except for a squamous or basocellular carcinoma of the skin, a prior radio(chemo)therapy treatment within the last six months, active intestinal co-morbidity or a known eating disorder precluding adequate dietary intake or absorption, medicinal use of anti-coagulants (e.g. warfarin, fenprocoumon, acenocoumarol, nadroparin (high dose >0.4 ml), enoxaparin (>40 mg) or tinzaparin) and/or anti-epileptics in case of history of epilepsy, diagnosis of heart failure, uncontrolled diabetes, (severe) dementia, pregnancy or lactation, use of *n*-3 FA supplements in the last 4 weeks prior to enrolment, known allergy for *n*-3 FA supplements and inability for adequate communication in Dutch or French. Patients were consecutively recruited from May 2012 until December 2013 upon presentation at the departments of radiotherapy at General Hospital Groeninge and Ghent University Hospital. Informed consent was obtained from all included patients. The trial was approved by the Ethics Committees of both hospitals.

### Study design

#### Randomisation

A multicentre, placebo-controlled, double-blind trial was performed. Randomisation was performed at time of enrolment to the control group (C) or the intervention group (I), by minimisation, a method of adaptive stratified sampling, as described by Pocock and Simon [24]. Gender, tumour stage, treatment plan and trial site were taken into account for equal allocation of patients. A random element was also incorporated to avoid predictability of the treatment allocation. An MS Office Excel macro, incorporating abovementioned characteristics, was created by the designated senior statistician (Prof. H. Pottel) for the convenience of the study coordinator, who was responsible for the central randomisation of patients.

#### Dietary supplements

Patients in the I group were asked to consume 7.5 ml *bis in die* (b.i.d., twice a day) (15 ml in total) of echium oil (BioMega SDA®, containing 235 ± 30 mg/ml ALA + 95 ± 13 mg/ml ALA SDA + 79 ± 10 mg/ml GLA) daily from treatment initiation, until the end of their 7-week treatment. Patients in the C group received a placebo, containing the same volume of *n*-3 PUFA deficient sunflower oil (Sunflower Oil High Oleic). The complete FA composition of both dietary supplements is described in Table 1. Bioriginal Food and Science corp. (Bioriginal Europe/Asia B.V., Den Bommel, The Netherlands) provided both dietary supplements in a liquid formulation packaged identically in sachets of 7.5 ml volume for convenience of the patient. Patients were allowed to take the supplements orally or via an enteral feeding tube if present. Due to the limited stability, dietary supplements were delivered in three different batches, each checked for FA composition consistency by the Department of Microbial and Molecular Systems (Catholic University Leuven Kulak) as determined by Ryckebosch *et al.* [25]. Letter-coded sachets were directly delivered by the supplier, and presented to the patient after randomisation by the study coordinator at time of first evaluation. Only the designated pharmacist of the coordinating centre possessed the information necessary for decoding, which was delivered in a sealed envelope by the supplier, and opened for unblinding after database lock and prior to statistical analyses.

**Table 1:** **Fatty acid composition of dietary supplements**

% Total fatty acid composition Name	BioMega SDA® (I group) mean ± SD	Sunflower oil high oleic (C group) mean ± SD
C16:0 Palmitic acid (P)	7.28 ± 0.17	4.00 ± 0.00
C18:0 Stearic acid (S)	3.90 ± 0.08	2.88 ± 0.05
C18:1 (*n*-9) Oleic acid (O)	15.35 ± 0.24	84.48 ± 0.05
C18:2 (*n*-6) Linoleic acid (LA)	16.13 ± 0.10	8.40 ± 0.00
C18:3 (*n*-3) Alpha Linolenic acid (ALA)	31.38 ± 0.25	-
C18:3 (*n*-6) Gamma Linolenic acid (GLA)	11.00 ± 0.00	-
C18:4 (*n*-3) Stearidonic acid (SDA)	12.60 ± 0.22	-
C22:1 (*n*-9) Erucic acid (EA)	-	-
Trans fatty acids	-	-

Abbreviations: I Intervention, C Control.

#### Compliance

Patients were asked to return all unconsumed sachets for calculation of total dietary supplement intake. In addition, compliance was verified by collection of a 4 ml EDTA anti-coagulated blood specimen obtained at baseline and during week 4 of therapy, at the same time of routine blood collection, for quantification of erythrocyte *n*-3 LC PUFA levels. EDTA blood samples were stored at 4°C until retrieval by the Cerba European Lab (Brussels, Belgium) the same day. There, total lipid fraction was removed and injected into the gas–liquid chromatograph for quantification, according to their in-house protocol. Results are presented as % of total fatty acid composition. All fatty acids eluting between myristic acid and docosahexanoic acid were determined.

#### Nutritional status, quality of life assessment and body composition

Body weight was measured weekly from the start of radiation treatment until treatment end. Patients were weighed before or after their irradiation, while wearing light clothing and no shoes. The same scale was applied for consecutive visits. Body weight loss, as the primary endpoint, was calculated as ((body weight_treatment start_ – body weight_therapy end_)/( body weight_treatment start_))*100.

For collection of secondary endpoint data, the study coordinator, assisted by a dietician specialised in oncology, assessed nutritional status of every patient at baseline (week 0, W0), mid-therapy (W4) and at treatment end (W7) by the Patient-Generated Subjective Global Assessment (PG-SGA), as this is considered the gold standard for evaluation of nutritional status in oncology [26–28]. Higher PG-SGA scores indicate worsened nutritional status. A score of 9 or higher implies “a critical need for improved symptom management and/or nutrient intervention options”. Additionally, all patients were asked to self-complete the EORTC quality of life core questionnaire (QLQ C30) and the H&N cancer specific (HN35) questionnaire at those three time-points. The C30 enables assessment of the generic aspects of quality of life (functional, physical, cognitive, emotional, social and nutritional status) of cancer patients, while the HN35 adds H&N cancer specific questions [29, 30]. As an estimate of usual dietary intake, patients were asked to complete a 3-day-food journal prior to each assessment. Mean total energy and macronutrient (carbohydrate, protein, fat, alcohol) intake were calculated with the Becel Institute Nutrition Software (BINS; Vodisys Medical Software, Groningen, The Netherlands). This software uses the Belgian (NUBEL, 1999) and Dutch (NEVO, 2001) food composition database. Body composition was assessed at W0 and at the end of W4, using both a bioelectrical impedance analyser (BIA 101, Akern S.r.I., Firenze, Italy), and Dual energy X-ray absorptiometry (DXA) as the gold standard. Bioimpedance was measured after a night’s fast, using a 50 kHz electrical signal of a 400 μA current traveling through source electrodes placed on the patient’s distal metacarpals while the patient was lying supine on an examination bed. Resistance, capacitive reactance and anthropometric values were entered in the Bodygram Pro® software (version 3.0, Akern, Italy) for calculation of fat mass (FM), fat free mass (FFM) and lean mass (LM). Patients were scanned with the Hologic Discovery Wi QDR Series (S/N 84566; fan-beam, switched pulse dual-energy x-ray tube, operating at 100 kV and 140 kV) or the Hologic Discovery A QDR series 4500A (S/N 45018; fan-beam) in the General Hospital Groeninge (AZG) and the Ghent University hospital (UZG) respectively. Data were analysed with the Apex System Software version 3.1.2 (AZG) or the Software version 12.7.3.1:3 (UZG) and collected in a database. A whole body DXA quality control phantom was scanned ten times on both appliances, for cross-calibration and calculation of a correction factor, enabling correct comparison of the data. Hand grip strength was measured with the JAMAR® hand dynamometer (Sammons Preston, Bolingbrook, IL, USA), as it is reflective of muscle mass [31].

Patient demographics, clinical characteristics, and side effects related to dietary supplements were collected by the study coordinator through the medical records, and registered by means of the ‘Common Toxicity Adverse Event (CTCAE v4.0)’ criteria.

#### Statistical analysis

Sample size calculations were based on the weight loss between baseline and end of therapy, of patients who were recruited in the OMGIANT study (oncogeriatric observational study in which older H&N cancer patients treated with curative intent were recruited, NKP_24_018) [32, 33]. In this study, an average weight loss of 5.46% (SD 4.63%) was reported for 38 patients (data not shown). The two-sided t test was used as a test statistic. The *null* hypothesis states that there is no difference between group averages (average weight loss). In order to show an (absolute) difference of 3% (i.e. the desired clinical effect) weight loss between the two groups at 80% power and 5% significance level, a sample size of 39 (evaluable) patients in each group was proposed. Prior to recruitment start, the trial was registered on ClinicalTrials.gov Identifier: NCT01596933. Body weight loss data of both groups as well as the between-group difference (effect size), as the primary endpoint, are expressed both as median (Q1, Q3) and mean ± SD, and 95% confidence intervals (95% CI) were calculated. Mann Whitney U statistics was applied to look for weight loss differences between both groups, according to the intention-to-treat principle. Secondary endpoint results are expressed as median (Q1, Q3) due to non-normality of the data. Mann Whitney U and Wilcoxon Signed Rank test were performed to explore respectively between- and within-group differences in fatty acid profile, body composition and grip strength. Spearman correlations were calculated to look for potential associations between supplement intake, *n*-3 LCPUFA changes and weight loss. Wilcoxon Signed Rank test was applied to explore within-group differences in nutritional status, caloric intake and quality of life during treatment, while repeated measures ANOVA models were established to calculate group differences of the latter parameters after correction for baseline values, age, gender, supplement intake, presence of artificial nutrition, tumour stage, tumour location and treatment. All analyses were performed using Prism® software (GraphPad Prism 5, Inc., La Jolla, CA) and SPSS or SAS software (version 20; IBM SPSS Statistics, Chicago, IL; SAS Institute Inc., Cary, NC, USA). Primary endpoint results were considered statistically significant at *P* < 0.05. Since the sample size calculation was only based on the primary endpoint hypothesis, all *P*-values obtained from secondary analyses should be considered as explorative, and thus have a merely suggestive value.

## Results

### Study population characteristics

Ninety-one eligible patients were enrolled and randomised, of whom 85 effectively started dietary supplement consumption. Prior to supplement intake, three patients declined radio(chemo)therapy and three others withdrew from study participation, one because of a relative who had advised against trial participation and two because of lack of understanding associated with non-compliance.

The study population comprised primarily married (76.5%), male (83.5%) patients, aged 61.0 years old (range 30 – 81), with an ECOG PS ≤1 (84.7%), and who were mostly working as an employee (45.9%). Most patients were current alcohol users (70.6%), with a median of 3 (1.0, 6.5) consumptions a day, and former smokers (57.6%) with a median of 30 pack years (14.3, 41.0). Patients predominantly presented with an advanced stage (III-IVb) cancer (83.5%) of the pharynx (57.6%), oral cavity (21.2%) and glottis (15.3%). Five (5.9%) patients presented with regional (lymph node) metastases of an occult primary. Approximately half of patients (48.2%) were treated with primary radiochemotherapy (Table 2).

**Table 2:** Demographic and clinical characteristics*

	Intervention group (I) N = 43	Control group (C) N = 42
**Demographic characteristics**	*Median (range)*	
Age	61.0 (30 – 81)	60.5 (35–79)
***Gender***	*n (%)*	
Male	36 (83.7)	35 (83.3)
Female	7 (16.3)	7 (16.7)
ECOG PS ≤1	34 (79.1)	38 (90.5)
***Social status***		
Married/living together	31 (72.1)	34 (81.0)
***Profession***		
Labourer	18 (41.9)	17 (40.5)
Employee	20 (46.5)	19 (45.2)
Self-employed	5 (11.6)	5 (11.9)
	*Median (Q1, Q3)*	
Alcohol consumption (U/day)	4.0 (1.0, 8.0)	3.0 (1.0, 6.3)
Tobacco consumption (PY)	25.5 (8.6, 40.3)	35.0 (17.8, 50.0)
**Clinical characteristics**	*n (%)*	
***Tumour location***		
Oral cavity	11 (25.6)	7 (16.7)
Oropharynx	17 (39.5)	17 (40.5)
Hypopharynx	7 (16.3)	4 (9.5)
Nasopharynx	2 (4.7)	2 (4.8)
Supraglottis	2 (4.7)	7 (16.7)
Glottis	2 (4.7)	2 (4.8)
Occult primary	2 (4.7)	3 (7.1)
***Tumour stage***		
Early stage (I-II)	6 (14.0)	8 (19.0)
Late stage (III-IVb)	37 (86.0)	34 (81.0)
***Therapy initiation***		
*Neoadjuvant* chemotherapy + radiochemotherapy	2 (4.7)	4 (9.5)
*Primary radiotherapy*		
Alone	7 (16.3)	8 (19.0)
With chemotherapy	22 (51.2)	18 (42.9)
With biotherapy	0 (0.0)	2 (4.8)
*Adjuvant radiotherapy*		
Alone	5 (11.6)	3 (7.1)
With chemotherapy	7 (16.3)	7 (16.7)
With biotherapy	0 (0.0)	0 (0.0)
**Nutritional characteristics**	*Median (Q1, Q3)*	
6-months weight loss (%)	2.5 (6.8, −1.5)	2.2 (5.4, 2.4)
BMI (kg/m^2^)	22.80 (20.50, 28.70)	24.25 (21.8, 27.2)
PG-SGA score	10.0 (5.0, 15.0)	8.5 (6.8, 13.0)
Caloric intake (kcal/day)	1784 (1449, 2284)	1759 (1531, 2198)

*Both groups did not significantly differ in demographic, nor clinical, nor nutritional characteristics at *P* < 0.05.

**Figure 1:**
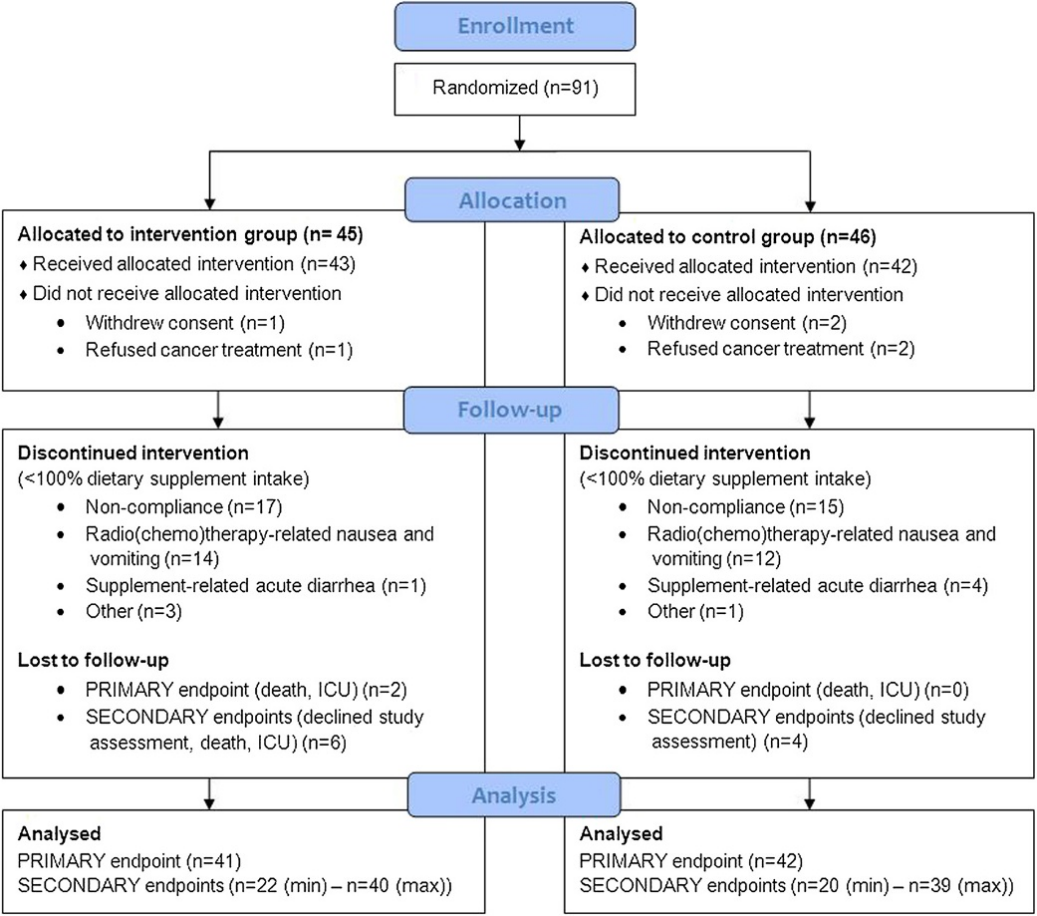
**Patient allocation flow diagram.**

At presentation, patients had a median 6-month-weight loss of 2.4% (6.3, −1.6), a median BMI of 23.9 kg/m^2^ (21.3, 27.5), and a median PG-SGA score, as an index for nutritional status, of 9.0 (6.0, 14.0). The median daily caloric intake was found to be 1781 kcal (1466, 2231). According to the definition of Fearon *et al.* [5] diagnosing patients as cachectic when a 6-month involuntary weight loss of 5% or more is present, 31% of study patients were considered cachectic at therapy initiation. There were no differences in baseline demographic, clinical or nutritional characteristics between the I and C group, except for a significantly lower DGLA fatty acid value in the I group (Table 2). Compliance to the study assessments was 89.4% (I: 90.7%, C: 88.1%). Of the 85 patients who initiated dietary supplement consumption, body weight loss was collected in 83 of them (I: 95.4%, C: 100%; primary endpoint), 77 patients were evaluated at W4 (I: 90.7%, C: 90.5%) and 76 (I: 90.7%, C: 88.1%) at therapy end for secondary endpoints. A patient allocation flow diagram is presented in Figure 1.

### Nutritional supplement tolerance

The median (Q1, Q3) supplement intake was 86.7% (30.2, 96.0) and 80.6% (43.6, 99.5) of the intended supplement intake for respectively the I and C group (p = 0.739) (Table 3). Both the liquid BioMega SDA® and the Sunflower oil High Oleic were equally well tolerated, with approximately three quarter of patients reporting good tolerance of the dietary supplement (resp. 72.1% vs 71.4%; p = 1.00). Although no serious adverse events (SAEs) related to the dietary supplements were reported, reasons for non-adherence were general non-compliance to the study protocol or forgetfulness (I: 48.6%, C: 46.9%), stomach ache, nausea, vomiting, and aversion for dietary supplement texture related to radiochemotherapy or strong opioid pain medication (I: 40.0%, C: 37.5%), and supplement-related acute diarrhea (I: 2.9%, C: 12.5%). Four patients stopped supplement intake because of respectively PEG-tube leakage (I: 0.0%, C: 3.1%), inability to swallow liquids after tracheotomy surgery (I: 2.9%, C: 0.0%), supplement-related appetite loss (I: 2.9%, C: 0.0%) and no further explained inability to swallow the oil (I: 2.9%, C: 0.0%).

**Table 3:** **Adherence to dietary supplements**

Supplement intake (%)	I group n = 43; n (%)	C group n = 42; n (%)
100%	8 (18.6)	10 (23.8)
≥ 75% - < 100%	17 (39.5)	16 (38.1)
≥ 50% - < 75%	6 (14.0)	4 (9.5)
≥ 25% - < 50%	2 (4.7)	3 (7.1)
< 25%	10 (23.3)	9 (21.4)

Abbreviations: I intervention, C control.

### Effect on weight loss (primary endpoint)

Of the eighty-five patients who effectively started nutritional supplement intake, weight loss data at therapy end were available for 83 patients. Missing data of two patients was attributed to respectively patient hospitalisation to the intensive care unit (where data were no longer collected) and patient death. According to the intention-to-treat principle, data of all 83 patients were included. No significant difference in weight loss was observed between the I group (n = 41, median (Q1, Q3); 8.9% (4.6, 11.8); mean ± SD (95% CI) 8.1 ± 5.4 [95% CI (6.5, 9.8)]) and the C group (n = 42, 7.6% (4.9, 9.8); 7.0 ± 5.1 [95% CI (5.4, 8.6)]) (p = 0.303). The body weight loss difference between both groups, or the effect size, was 1.13 [95% CI (−1.16, 3.42)]. A comparable percentage of patients in both groups required hospitalisation for artificial nutritional support (I: 18.6%, C: 26.8%, p = 0.439).

Spearman correlations only revealed a negative association (r_s_ = − 0.47, p < 0.01) between supplement intake and weight loss in the I group. Percentage weight loss was also found to negatively correlate with changes in ALA (r_s_ = − 0.42, p < 0.01), EPA (r_s_ = − 0.41, p < 0.01), and DGLA (r_s_ = − 0.37, p < 0.05) in the I group. In the C-group weight loss negatively correlated with GLA (r_s_ = −0.48, p = p < 0.01).

### Effect on erythrocyte fatty acid profile

Prior to nutritional supplement intake no significant differences were observed in the fatty acid profiles of both groups, except for a significantly lower DGLA value in the I group (Table 4).

**Table 4:** Nutritional supplement-related erythrocyte fatty acid profile changes (expressed as% total fatty acid composition; n = 79)*

Fatty acids	W0			W4			% change		
	I	C	p	I	C	p	I	C	p
***n*** **-3**									
**ALA**	0.14 (0.11, 0.20)^c^	0.13 (0.12, 0.18)^b^	NS	0.17 (0.14, 0.23)^c^	0.12 (0.09, 0.15)^b^	**<0.0001**	25.40 (−4.50, 63.60)	−16.70 (−33.30, 0.00)	**<0.0001**
**EPA**	0.66 (0.47, 0.81)^c^	0.68 (0.57, 0.88)	NS	0.75 (0.66, 0.90)^c^	0.62 (0.50, 0.82)	**0.02**	14.40 (−2.78, 37.78)	−5.30 (−21.80, 8.80)	**<0.001**
***n*** **-6**									
**LA**	6.72 (5.94, 7.49)^d^	6.71 (5.88, 7.44)^a^	NS	6.03 (5.25, 6.81)^d^	6.68 (5.71, 7.16)^a^	NS	−8.35 (−15.38, −2.48)	−3.80 (−9.60, 3.50)	**0.02**
**GLA**	0.04 (0.02, 0.05)^b^	0.05 (0.03, 0.06)^a^	NS	0.06 (0.04, 0.07)^b^	0.03 (0.02, 0.06)^a^	**<0.01**	41.65 (−20.00, 133.3)	−20.00 (−50.00, 16.70)	**<0.001**
**DGLA**	1.21 (1.13, 1.42)^d^	1.34 (1.21, 1.65)	**0.04**	1.52 (1.22, 1.78)^d^	1.36 (1.14, 1.68)	NS	18.75 (1.75, 35.30)	2.40 (−6.20, 6.80)	**<0.0001**
**AA**	12.84 (11.39, 13.35)	12.80 (11.72, 13.50)	NS	12.78 (11.77, 13.37)	12.71 (11.71, 13.64)	NS	2.35 (−2.05, 7.13)	−0.80 (−5.30, 5.00)	NS

*Data presented as median (Q1, Q3). Within-group significant differences were indicated by letters a (p < 0.05), b (p < 0.01), c (p < 0.001), d (p < 0.0001). Five patients refused second blood analysis, one was admitted to ICU. Significant *P*-values provided in bold.

**Figure 2:**
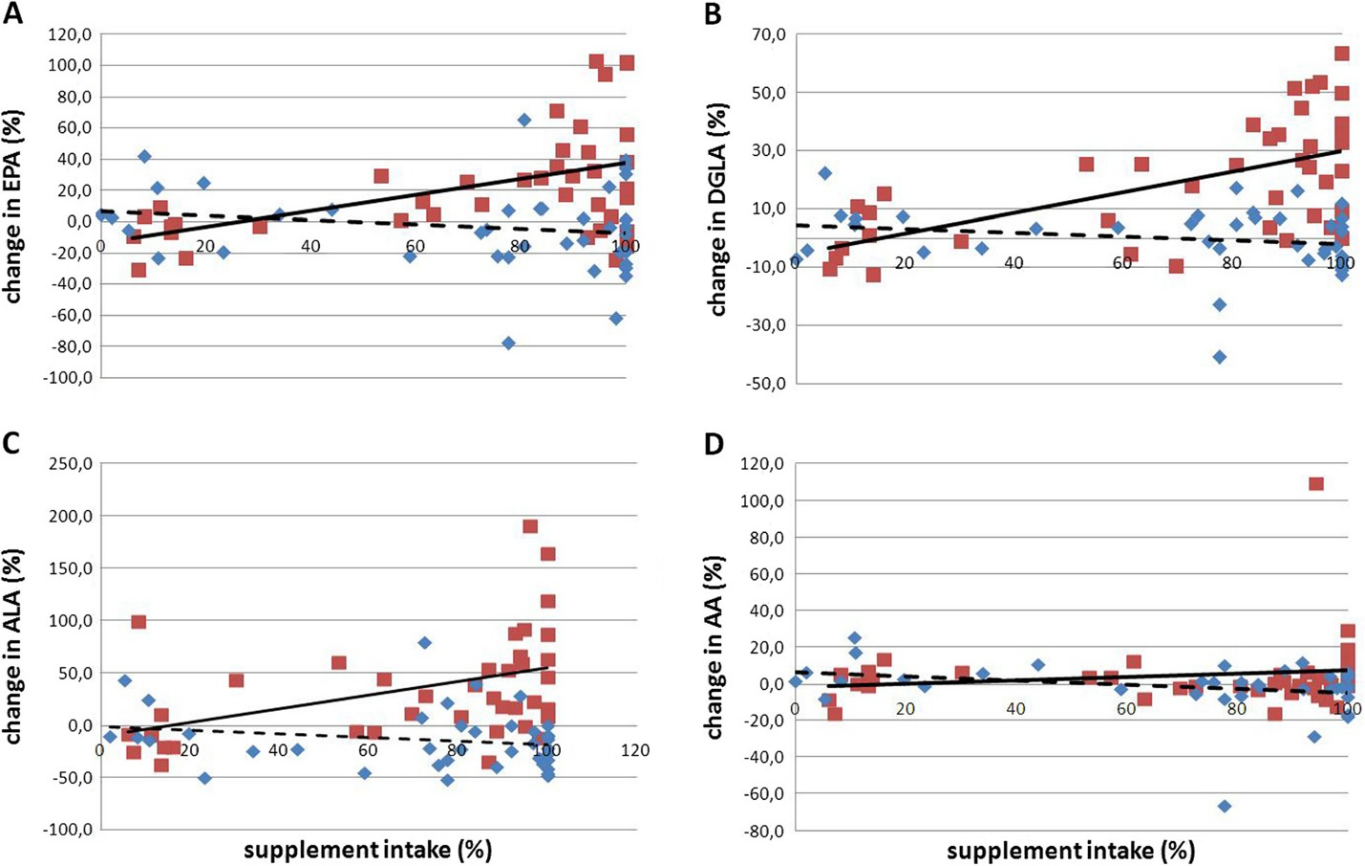
**Percent change in erythrocyte fatty acid profile after dietary supplement consumption.** The I and C group values are presented as respectively red squares and blue diamonds, and the resp. full and dashed line. **A**. Percent change in EPA in I (r 0.50, *P* < 0.01) and C (r −0.17, *P* > 0.05), **B**. Percent change in DGLA in I (r 0.60, *P* < 0.0001) and **C** (r −0.24, *P* > 0.05), **C**. Percent change in ALA in I (r 0.43, *P* < 0.01) and C (r −0.19, *P* > 0.05), and **D**. Percent change in AA in I (r 0.16, *P* > 0.05) and C (r −0.28, *P* > 0.05). EPA: eicosapentaenoic acid, ALA: α-linolenic acid, DGLA: dihomo-γ-linolenic acid, AA: arachidonic acid.

After 4 weeks, echium oil intake led to significant median increases in ALA (25.4%), EPA (14.4%), GLA (41.7%), DGLA (18.8%), a non-significant median increase in AA (2.4%), and a significant median decrease in LA (−8.4%) compared to baseline. In the C group median reductions in all FAs were seen, except for DGLA. The relative change over time of all FAs significantly differed between the I and C group, except for AA (Table 4). These changes resulted in significantly higher absolute values of ALA, EPA, and GLA in the I group compared to the C group, but comparable values of LA, AA and DGLA. The latter could be attributed to the significant lower DGLA concentration that was observed at baseline in the I group. In the I group, a dose–response relation was observed between the amount of dietary supplement ingested and changes in *n*-3 ALA (r_s_ = 0.51, p < 0.001), *n*-3 EPA (r_s_ = 0.39, p < 0.05) and *n*-6 DGLA (r_s_ = 0.57, p < 0.001) (Figure 2). No significant correlations were observed in the C group.

### Effect on other anthropometric parameters

No significant differences in baseline fat free mass (FFM), fat mass (FM), lean mass (LM), and grip strength were observed between both groups, assessed with DXA, BIA and JAMAR^®^ hand dynamometer respectively. After 4 weeks of dietary supplement intake, a significant decrease in FFM (I: median −5.10%, C: −4.12%, *P* < 0.01) and LM (I: −3.77%, C: −2.41%, *P* < 0.01) was observed in both groups with DXA. A respectively significant and borderline significant decrease in FM was observed in the I (−4.04%, *P* < 0.05) and C (−3.66%, *P* = 0.05) group. The relative change of FFM, LM and FM compared to baseline did not significantly differ between both groups. BIA measurement revealed a significant relative increase in FM in the I group, compared to the C group (10.55% vs −3.90%, *P* < 0.05). The relative median decrease in grip strength throughout treatment was comparable between both groups (I: −0.86% vs C: −0.78%, *P* > 0.05).

Prior to treatment start, both groups did not significantly differ in nutritional status or caloric intake. Radio(chemo)therapy had a significant negative impact on nutritional status, measured as PG-SGA, at W4 in both groups, and this remained stable until the end of treatment (data not shown). Reported dietary intake showed a decrease in caloric intake in both groups, however no statistically significant intervention effect was observed between the I and C group after correction for age, weight, length, gender, tumour location, tumour stage, and supplement intake (data not shown).

### Effect on quality of life and treatment-related toxicity

The percentage of severe-grade treatment-related toxicity was not significantly different in the I group, compared to the C group (51.2% vs 69.0%; p = 0.122). A comparable number of patients of respectively the I and C group required hospitalisation (I: 27.9%, C: 35.7%; p = 0.490). None of the SAEs that occurred were considered to be due to dietary supplementation but more likely to be attributed to therapy-related toxicity or disease progression.

Self-reported quality of life, assessed with EORTC QLQ C30 and HN35, was analysed through a repeated measures ANOVA model, in which baseline QOL status was incorporated as a covariate, as well as age, gender, tumour location, tumour stage, initiated treatment and supplement intake as potential confounders. No significant difference in function or symptom scores was observed between both groups (data not shown). A recurrent data pattern, however, demonstrating a positive relation between supplement intake and respectively nutritional status and EORTC QLQ function scores, as well as an inverse relation between supplement intake and EORTC symptom scores was observed irrespective of the study group (data not shown).

## Discussion

H&N cancer patients are a nutritionally vulnerable population at increased risk of developing cancer cachexia. Conventional nutritional support has at present not succeeded in reversing the cachectic state. Recently, *n*-3 LCPUFA EPA under the form of fish oil has demonstrated anti-inflammatory properties that improved nutritional parameters in cancer patients. In latest years, the decline of wild fish stocks in combination with the increased applicability of *n*-3 LCPUFA in different health domains has stimulated the search for alternative sources of *n*-3 LCPUFA. In the present study, a clear increase in *n*-3 ALA and EPA, as well as *n*-6 GLA and DGLA were observed after echium oil consumption, at the median dose taken, without a significant increase in AA. Nevertheless, the intervention proved ineffective in reducing weight loss during radio(chemo)therapy. Also, no significant improvement in preservation of lean mass, nutritional status, hospitalisation requirement and self-reported quality of life was observed. To our knowledge, this is the first trial to report the effects of a plant *n*-3 PUFA supplement in a cancer population. Echium oil has been suggested as a good alternative to fish oil for vegetarians [20], since it contains high levels of ALA and SDA, which are converted to EPA. Dietary intake of ALA and SDA results in a 0.2-8% and 17-30% conversion to EPA, respectively, depending on both the lipid fraction studied and the absolute ALA or SDA intake of supplements administrated [21, 34–37]. Moreover, the high GLA content is said to reinforce the anti-inflammatory effects produced by EPA, through its metabolite DGLA. At the dose volume taken median erythrocyte EPA content increased approximately 14%, with a maximum of 103% after 4 weeks, while a 40% increase in GLA lead to an approximate 20% increase in DGLA. Although the daily echium oil volume comprised 3.5 g ALA and 1.4 g SDA, the median EPA increase observed is lower than the 20% and 75% EPA increase reported in healthy subjects after respectively 1.5 g and 1.3 g SDA consumption daily for 3 and 4 weeks [21, 35, 38]. The large variability that is seen in FA changes over time, could be related to variations in supplement adherence as well as the significant decline in caloric intake in general, and lipid intake in specific, hampering *n*-3 PUFA uptake [39–41]. Also non-dietary factors could be involved, such as absorption, metabolism, and genetic and lifestyle determinants that are known to affect FA concentrations in human tissues [42]. Although controversial, alcohol and smoking status has been reported to negatively impact FA profile [43, 44], and higher BMI was recently described to be associated with lower relative and net increases in EPA [45].

Although a significant median increase was observed, EPA and DGLA concentrations might however still have been insufficient to reverse weight loss. Previous trials reported EPA increases of 200 to 900% over a period of 3 to 8 weeks to be associated with beneficial effects on nutritional parameters [46, 47]. The latter increases are mostly measured in plasma phospholipids, which are much more sensitive to the last dietary intake than red blood cells, and might therefore not be adequately compared. However, Arterburn et al. reported a strong correlation between plasma phospholipid and red blood cell EPA contents [42]. Based on conversion rates observed in healthy patients [35, 37], one could argue that consumption of the echium oil volume as applied in our trial would result into a daily EPA dosage that is approximately half of the minimum effective dosage of 1.5 g suggested in previous publications [48, 49]. However, we hypothesized that the additional DGLA increase might be sufficient to exert comparable effects as seen with fish oil, since attenuation of body weight loss and reversal of tumour-induced lipolysis has been demonstrated in mice fed a diet supplemented with SDA and GLA [50, 51]. Moreover, one clinical trial randomising patients with acute lung injury between standard iso-nitrogenous and isocaloric enteral nutrition alone or combined with EPA and GLA for a period of 14 days showed improved oxygenation, and higher BMI in the experimental group [52]. According to the current published conversion rates [35, 37], the required daily echium oil volume intake would significantly have to increase in order to obtain comparable dosages of EPA as found in fish oil (e.g. 2.4 g EPA would require 60 ml echium oil). However, this might still not show the intended EPA increase since diets containing above-optimal levels of *n*-3 PUFA might not lead to a further increase in EPA in humans, given that the most efficient conversion rates are on *n*-3 PUFA deficient diets [53]. At the median dose taken, cases of abdominal discomfort and belching have been reported, although they were mostly attributed to chemotherapy-related general nausea resulting in an aversion for the dietary supplement texture. Intake of larger volumes of echium oil might not be feasible under radiochemotherapy conditions. Furthermore, increased volumes of echium oil are associated with larger GLA dosages. The current estimated EPA:GLA ratio of 0.67:1 did not show a significant increase in AA at week 4, which is in agreement with Geppert et al. reporting preservation of AA values after GLA consumption in a EPA:GLA ratio of 0.2:1 [54]. However, to avoid a negative shift towards increased *n*-6 LCPUFA AA values, GLA dosage was kept within acceptable ranges [55].

Although progressive weight loss seen in our trial is comparable to non-intervention trials in H&N cancer patients reporting mean weight losses of 5.7% to 10%, Spearman correlations are suggestive for a positive effect of ALA, EPA and DGLA on weight loss. Prior trials reporting improved nutritional status related to EPA in H&N and esophageal cancer patients provided *n*-3 LCPUFA enriched protein- and energy-dense nutritional supplements for approximately 4 to 12 weeks peri- or postoperatively to improve caloric intake and stimulate lean mass anabolism [11, 13, 14]. Since in our trial oral nutritional supplementation was presented at time of weight loss initiation, and since it is difficult to differentiate whether the ongoing weight loss is related to tumour- or treatment-related dysphagia rather than anorexia or systemic inflammation due to metabolic changes, it could be possible that underlying starvation mechanisms might have limited the effectiveness of the current intervention [56]. However, although a significant decline in caloric intake – comparable with literature [57, 58] – was observed, no significant differences in caloric intake that could have had an impact on weight loss were seen between both groups. Moreover, the international consensus definition that was established for cancer cachexia by Fearon et al. does not suggest an alternative definition for both H&N or esophageal cancer, in which reduced food intake secondary to dysphagia is common [5]. Although prophylactic energy-dense nutritional supplementation is not standard, it might have improved the tolerance of dietary supplements and retained caloric intake potentially reducing weight loss during intensive radio(chemo)therapy, since four trials were recently published that showed (tendencies towards) improvement in body cell mass [59], body weight [60, 61], and metabolic, inflammatory and oxidative stress parameters [62] in H&N cancer patients under radio(chemo)therapy after administration of prophylactic enteral nutrition or high caloric oral nutritional supplements enriched with *n*-3 PUFA, and specific micronutrients or probiotics.

The significant decrease in lean mass seen in both groups, could be attributed to the decline in protein intake. The relative increase in fat mass, as measured by BIA was, however, not confirmed by DXA, which was used as gold standard. Since FM is calculated from FFM, this increase could be relative to the decrease in FFM seen in these patients. Moreover, dehydration is common in H&N cancer patients under radiochemotherapy, and can lead to an overestimation of fat mass measurements by BIA [63, 64]. PG-SGA scores, as a measure of nutritional status, increased equally throughout treatment in both groups, as did EORTC symptom scores. EORTC function scores decreased significantly at each time-point. This is in line with prior reports by our own research group [33]. Remarkably, a comparable “effect” of supplement intake on PG-SGA scores, EORTC function scales, and symptom scales ‘dry mouth’, ‘general feelings of illness’, ‘nausea and vomiting’ and ‘sticky saliva’ in specific, was seen in both groups. I.e., patients with a dietary supplement intake of 75% or more showed lower PG-SGA and EORTC symptom scores, while higher EORTC function scores compared to those consuming less than 75% of the supplements. Since these results are corrected for parameters influencing treatment toxicity, this could suggest that patients with the best understanding of the severity of their disease are the most motivated and treatment-compliant, and thus also the ones most adherent to the dietary supplement.

Our study results should be interpreted with caution due to some study limitations. First, the typical western diet already contains high levels of especially *n*-6 PUFA (ratio *n*-6/*n*-3 is 20:1) [65]. However, since we could not control the type of dietary fat in the background diet, this could be partly responsible for the strong variations seen in fatty acid profile at week 4. Also, patients with a percutaneous endoscopic gastrostomy (PEG) tube were not excluded from trial participation. Moreover, several patients required PEG-tube placement during therapy because of prolonged weight loss, and enteral nutrition can sometimes be enriched with *n*-3 LCPUFA. The number of patients with a prophylactic PEG-tube, or requiring one during treatment, did not significantly differ in both groups. Moreover, subgroup analyses excluding patients fed with enteral or parenteral nutrition were not suggestive for a change in trial results (data not shown). Second, four weeks of high oleic sunflower oil consumption (10.4 g oleic acid/day) seemed to lead to decreased values of both *n*-3 and *n*-6 PUFA, except for DGLA, which could be responsible for the significant difference in (LC)PUFA concentrations between both groups. This is not in agreement with literature, reporting comparable *n*-3 LCPUFA at both time points in the placebo group in non-small cell lung cancer patients during multimodality treatment [66, 67]. Indeed, this decline could be related to the strong decrease in caloric intake that is inherently seen in H&N cancer patients during treatment, since administration of 8.5 g high oleic sunflower oil over a 12-week period, did not change (LC)PUFA concentrations in a healthy population with a stable energy intake [34]. Moreover, Dessi et al. reported that nutritional deprivation in hemodialysis patients was associated with decreased PUFA abundance [39]. Subgroup analysis of those patients consuming less than 25% of the dietary supplement, were also suggestive for median decreased LCPUFA values (data not shown). Third, non-adherence to the dietary supplement did not lead to exclusion since the primary endpoint was analysed according to the intention-to-treat principle for which our trial was sufficiently powered. This was different than a publication by Murphy et al. who excluded patients with a supplement intake of less than 80% [66]. Patient adherence to the nutritional supplements was only moderate, however comparable in both groups. H&N cancer patients are known to lack motivation to comply with treatment or supportive care associated with it, and many will not complete clinical trials impeding accurate trial results [57, 68]. Comparable recurrent PG-SGA and EORTC QOL data patterns associated with supplement intake in both groups, confirm the potential bias created by patient non-adherence. Administration of echium oil already at time of diagnosis or as of radio(chemo)therapy end, may possibly increase patient adherence potentially leading to higher erythrocyte EPA increases and associated anti-inflammatory effects, and might therefore be worth exploring in the future. Fourth, we did not measure any inflammatory markers that could indicate if the median dose volume consumed did in fact improve systemic inflammation. Retrospective analysis of raw C-reactive protein (CRP) data collected during routine blood analyses at W4 showed median CRP increases in both groups during treatment (n = 60, I: 4.5 (−0.0, 12.0) and C: 1.4 (−2.0, 11.7)), while no significant difference in absolute CRP change was observed between both groups (*P* = 0.40). Last, we did not correct for multiple testing through application of the ‘α spending function’, since this correction primarily applies to multiple primary endpoints and since this would be extremely difficult considering the multitude of different statistical tests employed [69]. All secondary endpoint results are, therefore, to be considered as merely suggestive.

## Conclusions

In conclusion, echium oil supplementation was feasible, well-tolerated and – despite moderate adherence – intake resulted in the anticipated increase of erythrocyte EPA and DGLA, without a significant increase in AA. Echium oil, however, failed to protect against radio(chemo)therapy-induced weight loss in our study, and should therefore not yet be recommended in daily practice, outside of the context of a clinical trial.

## Abbreviations

AA: Arachidonic acid
ALA: α-linolenic acid
BIA: Bioelectrical impedance assessment
BMI: Body mass index
C30: Core 30
CRP: C-reactive protein
DGLA: Dihomo-γ-linolenic acid
DXA: Dual x-ray absorptiometry
ECOG PS: Eastern cooperative oncology group performance status
EORTC: European organisation for the research and treatment of cancer
FA: Fatty acid
FFM: Fat free mass
FM: Fat mass
GLA: γ-linolenic acid
H&N: Head and neck
HN35: Head and neck 35
LA: Linoleic acid
LC: Long chain
LM: Lean mass
N: Omega
PEG: Percutaenous endoscopic gastrostomy
PG-SGA: Patient generated-subjective global assessment
PUFA: Polyunsaturated fatty acid
QLQ: Quality of life questionnaire
QOL: Quality of life
SAE: Severe adverse event
SDA: Stearidonic acid.
